# Integrating Dynamic Representation and Multi-Priors for Transnasal Intubation via Visual Foundation Model

**DOI:** 10.3390/bioengineering13020217

**Published:** 2026-02-13

**Authors:** Jinyu Liu, Yang Zhou, Ruoyi Hao, Mingying Li, Yang Zhang, Hongliang Ren

**Affiliations:** 1 Hubei Key Laboratory of Modern Manufacturing Quality Engineering, Hubei University of Technology, Nanhu Avenue 28, Wuhan 430068, China; 102300032@hbut.edu.cn (J.L.); 102200036@hbut.edu.cn (M.L.); 2 School of Mechanical Science and Engineering, Huazhong University of Science and Technology, Luoyu Road 1037, Wuhan 430074, China; yzhoucst@hust.edu.cn; 3 Department of Electronic Engineering, The Chinese University of Hong Kong, Shatin, Hong Kong 999077, China; 1155167067@link.cuhk.edu.hk; 4 Key Laboratory of Symbolic Computation and Knowledge Engineering, Ministry of Education, Jilin University, Changchun 130012, China; 5 National Key Laboratory for Novel Software Technology, Nanjing University, Hankou Road 22, Nanjing 210093, China

**Keywords:** dynamic representation, low-rank constraint, visual foundation model, glottis segmentation

## Abstract

Accurate and real-time glottis localization is critical for ensuring intraoperative oxygenation and patient safety during nasotracheal intubation. However, representative foundation models exemplified by the Segment Anything Model exhibit notable limitations in medical applications, stemming from their rigid attention mechanisms, feature space misalignment, and insufficient generalization to complex glottal anatomies. To address these challenges, we propose Glottis-SAM, a lightweight and task-adaptive segmentation framework that integrates dynamic representation learning with multi-prior contextual modeling. Specifically, we introduce a hierarchical low-rank adaptation strategy that enables efficient fine-tuning of visual foundation models by preserving geometric priors while significantly reducing computational overhead. To further enhance semantic fusion and generalization, we design a feature aggregation module with dual-path dynamic feature pyramids, which enables complementary optimization from local textures to global semantic structures under varying anatomical conditions. Extensive experiments on three diverse datasets demonstrate that Glottis-SAM achieves state-of-the-art segmentation accuracy with 72.6% mDice, a compact 55.2 MB model size, and 44.3 FPS inference speed on clinical data. These results highlight the model’s robustness, efficiency, and potential for deployment in visual guidance systems for nasotracheal intubation.

## 1. Introduction

Medical image segmentation, as a core component of computer-aided diagnosis systems, plays a pivotal role in precisely characterizing anatomical structures and pathological features from multimodal medical images [1]. Among various clinical procedures, nasotracheal intubation (NTI) presents unique challenges for real-time anatomical localization. Inaccurate or delayed identification of the glottis during NTI can lead to severe complications, including hypoxemia, tracheal injury, and hemodynamic instability, especially in patients with difficult airways or in emergency settings [2]. Currently, clinical tracheal intubation is primarily performed by anesthesiologists, critical care physicians, or emergency clinicians using visual laryngoscopy [3]. The success of the procedure strongly depends on the operator’s experience and technical proficiency. To improve the accuracy, efficiency, and standardization of NTI, the integration of computer-aided diagnosis technologies and intelligent medical systems has emerged as a promising direction in clinical practice. Unlike conventional static segmentation tasks, NTI necessitates real-time localization of highly deformable and dynamic glottal structures under intricate clinical conditions. Key visual perturbations arise from motion-induced blur due to endoscopic movement, inconsistent illumination within confined anatomical cavities, and substantial inter-patient anatomical variability. These factors collectively impose stringent requirements on segmentation models, which must ensure not only high accuracy but also computational efficiency and robustness across diverse patient anatomies.

Traditional glottis segmentation methods [4] fundamentally rely on low-level image statistics. Due to the absence of anatomical semantics, they struggle to differentiate glottal boundaries from spatially adjacent structures, such as deformed vocal folds. CNN-based architectures have improved robustness through hierarchical feature learning; however, single-stage models typically compromise the accuracy of small targets to gain speed, while two-stage instance segmentation networks often lose edge details of large targets and introduce latency spikes, rendering them unsuitable for real-time NTI. Given these limitations, there is an urgent need for more robust and adaptable segmentation approaches that are capable of meeting the complex demands of clinical NTI scenarios. A recently proposed general-purpose vision segmentation foundation model, the Segment Anything Model (SAM) [5], has gained widespread recognition for its strong cross-object segmentation capability and zero-shot generalization performance. It can identify and segment objects based on user prompts, including points, bounding boxes, and coarse masks. Despite its success in natural image domains, SAM exhibits significant limitations in medical applications. The fundamental differences in imaging modalities, contrast distribution, and feature spaces often result in unstable segmentation across medical datasets [6]. Moreover, in vivo environments are characterized by weak boundaries, low contrast, and irregular structural deformations. Specifically, the dynamic opening and closing of the glottis varies dramatically in scale, which conflicts with SAM’s fixed-size window attention mechanism and hinders fine anatomical parsing.

**Figure 1:**
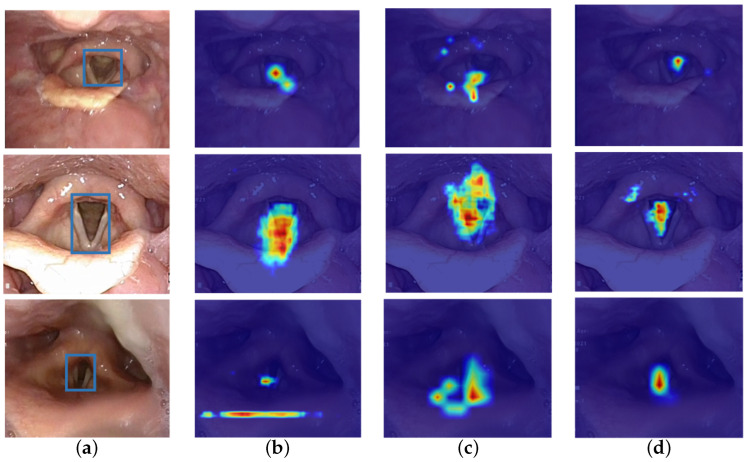
Visual comparison of activation map outputs across different model architectures. (**a**) Reference ground-truth labels (highlighted in blue) for each medical image. (**b**) Mask R-CNN predictions. (**c**) SAM predictions. (**d**) Glottis-SAM predictions. The color scale represents feature activation intensity, ranging from blue (low) to red (high). The comparative activation patterns demonstrate that our proposed approach achieves superior localization accuracy for the target anatomical regions.

As illustrated in Figure 1, SAM tends to produce spatially diffuse and structurally inconsistent attention maps when applied to glottic regions. In contrast, our proposed Glottis-SAM model produces more compact and precisely localized activation maps that better conform to the glottic region. Additionally, the default input resolution of SAM leads to excessive computational resource consumption, which exceeds the capacity of most endoscopic devices. These technical constraints, coupled with feature space misalignment, significantly hinder SAM’s direct clinical applicability and raise concerns regarding its deployment in real-time surgical scenarios.

In this paper, we present Glottis-SAM to transfer the exceptional segmentation performance and strong generalization ability of SAM to glottis image segmentation while reducing computational complexity. Our encoder effectively captures rich spatial contextual information to enable multi-scale context feature modeling. We propose a low-rank adaptation fine-tuning strategy, where learnable low-rank matrices are introduced to progressively adapt the hierarchical features of SAM while preserving its pretrained geometric priors and significantly reducing the number of trainable parameters compared to full fine-tuning. Furthermore, we design a feature aggregation module that dynamically calibrates feature responses via channel attention, establishes a continuous representation of spatial anatomical transformations, and promotes cross-layer feature complementarity. A dual-path dynamic feature pyramid is constructed to adaptively model multi-scale geometric features, incorporating multi-head collaboration under dynamic spatial constraints to achieve effective feature decoupling. Notably, comprehensive evaluations on the Glottis, Phantom, and Clinical datasets demonstrate that our model outperforms the state-of-the-art methods. The size of our tiny version model is only 55.2 MB. The primary contributions of this work include:

- Dynamic representation and adaptation of multi-scale glottal image features to precisely parse local details and global semantic priors, enhancing robustness to deformation and motion blur.
- A high-efficiency fine-tuning strategy that extends low-rank adaptation for hierarchical feature adaptation, preserving geometric priors while reducing computational overhead.
- A feature aggregator that dynamically calibrates feature responses and constructs a dual-path dynamic feature pyramid by incorporating attention-based adaptive fusion of multi-scale features, which improves generalization under complex internal cavity anatomies.

## 2. Related Work

### 2.1. Glottal Detection and Segmentation

Accurate localization of the glottis is essential in robot-assisted NTI as it supports clinicians in precise regional assessment and provides critical guidance for subsequent surgical procedures. The early approaches primarily relied on thresholding and region-growing algorithms. For instance, Cerrolaza et al. [7] proposed an adaptive seeded region-growing method with dynamic edge constraints to detect the glottal gap. Karakozoglou et al. [8] introduced a vibration-mode-coupled active contour model to improve segmentation stability under noisy conditions. However, these traditional techniques are highly sensitive to grayscale distributions and perform poorly in the presence of complex biological contamination.

With the rapid advancement of deep learning, CNN-based segmentation methods have become increasingly prominent in medical image analysis. Fehling et al. [9] introduced a hybrid architecture for dynamic glottis segmentation, incorporating temporal modeling across video sequences to enhance temporal consistency. To address the light scattering artifacts commonly observed in endoscopic imagery, Deng et al. [10] proposed an improved CycleGAN framework with phase consistency constraints, effectively suppressing sputum interference and improving the peak signal-to-noise ratio. Classical two-stage instance segmentation frameworks, such as Mask R-CNN [11], PointRend [12], MS-RCNN [13], and QueryInst [14], have shown promise by leveraging region of interest (RoI) alignment for precise feature refinement. However, these methods often suffer from significant feature degradation near object boundaries, particularly for large anatomical targets, limiting their applicability in dynamic glottis monitoring. In contrast, one-stage methods, including YOLACT [15], BoxInst [16], CondInst [17], SOLOv2 [18], RTMDet [19], and SparseInst [20], offer higher inference speed and real-time capabilities. Yet, their segmentation accuracy on small or occluded structures tends to degrade due to the absence of explicit positional priors and fine-grained boundary supervision. In parallel, the U-Net architecture remains a foundational design in medical image segmentation and has been widely applied to glottal region analysis. Extensions such as U-Net++ [21] and U-Net 3+ [22] have improved multi-scale feature fusion, enhancing sensitivity to fine anatomical details. A subsequent study [23] validated the effectiveness of these models in glottis segmentation. Nonetheless, their limited representational capacity and reduced robustness under noisy or anatomically variable conditions constrain their performance in real-world clinical settings.

Despite recent progress laying the groundwork for robot-assisted glottis localization, several critical challenges remain. These include limited adaptability to glottal shape variations and imaging artifacts, stringent latency constraints, and inconsistent semantic prediction of segmentation masks under clinical conditions.

### 2.2. Foundation Model Adaptation for Medical Image Segmentation

In recent years, extensive efforts have been devoted to adapting general-purpose vision architectures for medical image segmentation, where challenges such as clinical datasets, anatomical variability, and domain shifts are prevalent. Several studies focus on enhancing architectural efficiency and semantic consistency. Chen et al. [24] proposed contextual and structural similarity losses to enforce semantic coherence across voxels. Huang et al. [25] introduced a hybrid network featuring dynamic positioning attention and bilevel routing attention, which improves both local detail extraction and computational efficiency. Task-specific frameworks such as RASNet [26] leverage multi-scale spatial perception and attention-based decoding for renal image segmentation. Meanwhile, the emergence of foundation models such as SAM sparked a surge of interest in harnessing their zero-shot segmentation capabilities for clinical applications. However, SAM’s performance often degrades significantly in medical imaging tasks due to challenges such as heterogeneous imaging modalities, indistinct tissue boundaries, and complex pathological variations. To address this technical bottleneck, researchers proposed various adaptations of SAM tailored for medical imaging. Regarding model fine-tuning strategies, MedSAM [27] introduced an innovative dual-encoder freezing mechanism, where both the image and prompt encoders were kept fixed and only the segmentation decoder was trained to adapt to medical scenarios. Silva et al. [28] proposed a few-shot efficient fine-tuning strategy, which enabled efficient adaptation of foundation models to few-shot medical image segmentation tasks through parameter-efficient tuning and black-box adapters. Wen et al. [29] employed a dual-branch architecture to extract critical medical semantic information and adopted a hierarchical fusion strategy, demonstrating strong performance across various multimodal medical image fusion tasks.

Moreover, recent studies have increasingly focused on integrating medical prior knowledge into model architecture design. PFEMed [30] introduced a dual-encoder framework combining general visual priors with task-specific representations, further enhancing feature robustness via a prior-guided variational autoencoder. Zhao et al. [31] introduced a novel Prior Attention Network that adopted a coarse-to-fine strategy for multi-lesion segmentation in medical imaging. Shi et al. [32] proposed a mask-enhanced adaptive encoder that incorporated multi-scale feature fusion and a lesion-aware attention module. You et al. [33] developed a novel shape prior module that explicitly integrated both global and local shape priors to enhance the segmentation performance of UNet-based models. Inspired by the versatility of foundation models, we propose Glottis-SAM, which integrates task-critical and dynamic contextual priors to learn image embeddings from glottis images. To enable efficient fine-tuning, we adopt a low-rank adaptation (LoRA) strategy [34]. The model focuses on multi-scale feature learning and reduced computational cost, aiming for practical deployment in clinical NTI applications.

## 3. Methods

**Figure 2:**
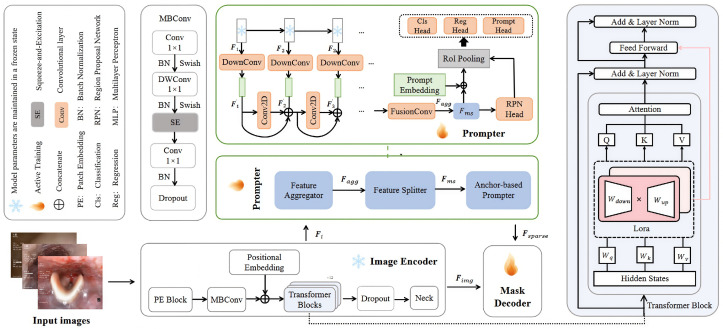
Overview of the Glottis-SAM framework for glottis segmentation in nasotracheal intubation. A frozen image encoder extracts deep semantic features $F_{\mathrm{img}}$ and intermediate representations $F_{i}$ from input images. $F_{\mathrm{img}}$ is forwarded to a multi-prior-guided decoder for initial mask prediction, while $F_{i}$ undergoes hierarchical low-rank adaptation via LoRA to refine multi-scale features. Channel attention is applied to produce aggregated feature $F_{\mathrm{agg}}$, which is structured into a dynamic dual-path feature pyramid for robust semantic modeling across varying anatomical scales. Finally, prompt vectors derived from semantic features are passed to the SAM decoder to generate accurate real-time glottis masks.

We propose Glottis-SAM, a dynamic segmentation framework that integrates multi-scale features and task-specific priors. As shown in Figure 2, the framework comprises a parameter-efficient fine-tuned encoder for multi-scale feature extraction, a dynamic multi-prior feature aggregation prompter for cross-layer complementary optimization, and a decoder.

### 3.1. Multi-Scale Contextual Feature Encoding

In medical tasks involving NTI, imaging-guided navigation systems must accurately identify and localize complex anatomical structures, including nasal morphology, pharyngeal curvature, and the precise position of the trachea, while simultaneously meeting the clinical demand for real-time dynamic responsiveness. Significant variability across patients further exacerbates the challenges for achieving high segmentation accuracy and effective multi-scale feature extraction. SAM is built upon a transformer architecture, and its encoder inherits a powerful self-attention mechanism that effectively captures long-range dependencies and rich spatial contextual information. Its efficient feature encoding and boundary modeling capabilities allow for precise delineation of anatomical structures, which is crucial for identifying key landmarks during intubation. However, due to the computational constraints of medical hardware, direct deployment of SAM remains impractical in real-world clinical settings. To address this issue, we adopt a Vision Transformer (ViT) backbone as the encoder, which offers a favorable balance between representation capacity and computational cost under constrained medical hardware. By adjusting the scaling factors $\left\{\zeta_{1},\zeta_{2},\zeta_{3},\zeta_{4}\right\}$ that control the embedding dimensions at each stage, we construct encoder variants with different capacities, enabling flexible trade-offs between accuracy and efficiency. The encoder captures both local texture and global structure, thereby enhancing the representation of glottal features. Overall, this design supports robust multi-scale contextual feature modeling in clinical environments with limited computational resources.

Given a glottis image $I\in R^{H\times W\times C}$, it is first processed by a patch embedding module composed of two consecutive 3×3 convolutional layers, each configured with a stride of 2 and padding of 1. This design facilitates hierarchical feature extraction and spatial downsampling, resulting in feature maps of size $\frac{H}{4}\times \frac{W}{4}\times C$, which are suitable for efficient representation learning. As shown in Table 1, ViT adopts a hybrid architecture that integrates MBConv and transformer blocks. Adjustable scaling factors $\left\{\zeta_{1},\zeta_{2},\zeta_{3},\zeta_{4}\right\}$ control the embedding dimensions at each of the four stages. ViT-B uses $\left\{96,192,384,576\right\}$, while ViT-T uses $\left\{64,128,160,320\right\}$, enabling flexible control over the model’s capacity and computational cost.

In the initial stage, MBConv performs downsampling and leverages a bottleneck design to reduce computational complexity while effectively encoding low-level features. In the subsequent three stages, transformers equipped with a window-based self-attention mechanism perform deep feature extraction and multi-scale feature fusion $F_{ms}$. This localized attention strategy retains the modeling benefits of self-attention while significantly lowering computational overhead. Specifically, $\left\{F_{1},F_{2},...,F_{M}\right\}$ denote the multi-scale feature maps extracted from different transformer stages. Each feature map $F_{j}$ is transformed by a mapping function T(·) and weighted by a learnable coefficient $\omega_{j}$ to obtain the fused representation $F_{ms}$. Here, T(·) denotes a lightweight alignment operator that projects each $F_{j}$ to a unified channel dimension and resizes it to a common spatial resolution so that features from different stages can be aggregated by summation.

(1) $$F_{ms}=\sum_{j=1}^{M}\omega_{j}\cdot T\left(F_{j}\right).$$

To further enhance the perception of local structural information, a 3×3 depthwise-separable convolutional layer is inserted between the self-attention module and the multi-layer perceptron. This hybrid architectural design exploits the advantages of convolutional operations in local feature extraction, thereby improving the model’s representational capacity. By integrating multi-scale feature extraction strategies with a hybrid computational paradigm, the model achieves efficient and robust visual representation learning under constrained computational resources.

### 3.2. Hierarchical Low-Rank Adaptation for Efficient Fine-Tuning

Achieving task-specific adaptation for glottal features without compromising the global visual representation capabilities of a pretrained model remains a fundamental challenge in glottal image analysis. Although traditional full fine-tuning yields strong performance in downstream tasks, the billion scale parameters of the SAM model impose substantial computational and memory burdens, resulting in superlinear growth in resource consumption and hindering practical deployment where accuracy and efficiency must be balanced. Recent studies have shown that low-rank representation offers a promising solution to balance efficiency and performance. Inspired by LoRA [34], we propose a hierarchical low-rank adaptation mechanism for parameter-efficient fine-tuning. Specifically, we model the weight update in a factorized low-rank form using two trainable matrices, which enables efficient adaptation of high-dimensional weights with a small number of additional parameters. By extending LoRA across layers, the model learns task-specific representations while preserving the strong geometric priors of SAM, thereby improving its modeling capability and adaptability for glottal image analysis. The LoRA rank is selected based on ablation studies and detailed analysis, and the final configuration is provided in Section 4.4.2.

Specifically, we freeze the transformers to maintain fixed parameters $W\in R^{d\times d}$. This approach preserves the structural inductive biases learned from large-scale natural image datasets, particularly those that capture geometric priors such as edge continuity, spatial layout, and anatomical symmetry, while improving the model’s ability to adapt to specific glottal features. Simultaneously, we introduce trainable low-rank matrices into the query–key–value (QKV) projection matrices of the self-attention mechanism and the two fully connected layers of the feedforward network ($FC_{1}$, $FC_{2}$). The low-rank update is defined as ΔW=B·A, while $B\in R^{d\times r}$ and $A\in R^{r\times k}$ are learnable matrices. Only these localized parameters are updated, and the changes can be efficiently captured using a small number of principal components. The updated weights are formulated as $W^{\prime }=W+\Delta W=W+\frac{\alpha }{r}\cdot BA$, where *r* denotes the rank of the low-rank matrices used for normalization, and α is a scaling factor controlling the magnitude of BA.

By incorporating low-rank updates into the QKV projections, the model is able to learn attention patterns that are more specific to glottal structures. The attention computation is modified as follows:

(2) $$A^{\prime }=\mathrm{Softmax}\left(\frac{\left(Q+BA_{q}\right){\left(K+BA_{k}\right)}^{T}}{\sqrt{d}}\right)\left(V+BA_{v}\right).$$

This modification enhances the sensitivity to task-specific anatomical features while maintaining its general visual understanding capabilities. Additionally, LoRA within the feedforward network reinforces the nonlinear representation of glottal features. By constraining parameter updates to a low-dimensional subspace, the model is encouraged to learn essential and robust representations rather than overfitting to spurious patterns or noise. From a clinical deployment perspective, this lightweight adaptation strategy significantly reduces hardware requirements and improves inference efficiency, making it well suited for real-time applications in resource-constrained medical environments.

### 3.3. Dynamic Semantic Feature Aggregation

#### 3.3.1. Feature Aggregation

To further construct an efficient multi-scale semantic representation system, we design a feature aggregation module that employs a structured feature interaction mechanism to extract discriminative cross-scale semantic information from the intermediate features $F_{i}\in R^{H\times W\times C}$ generated by the backbone network. For each feature map $F_{i}$, the feature aggregate module first applies differential spatial scaling to dynamically align spatial dimensions. A 1 × 1 convolution is used for channel compression, which significantly reduces computational complexity while preserving critical discriminative information. This is followed by a 3 × 3 convolution for local receptive field enhancement, allowing the model to better capture spatial context. To further improve feature extraction efficiency, batch normalization is applied after the convolutional operations.

(3) $$F_{i}^{\prime }=P\left({\mathrm{Conv}}_{1\times 1}\left(F_{i}\right)\right)\in R^{H^{\prime }\times W^{\prime }\times C^{\prime }},$$

(4) $$F_{i}^{\prime \prime }=\sum_{d\in 1,2,3}\mathrm{BN}\left(\sigma \left(W_{3\times 3}^{d}{*}_{d}F_{i}^{\prime }+b\right)\right),$$

where P(·) denotes a parameterized downsampling operator, $W_{3\times 3}^{d}$ represents the convolution kernels with dilation rate *d*, and ${*}_{d}$ represents the dilated convolution operation. Subsequently, residual connections and element-wise addition are applied for adaptive fusion of multi-level features. The resulting temporary feature map is computed as

(5) $$F_{\mathrm{temp}}=\sum_{i=1}^{n}\varphi_{i}⊙\left(F_{i}^{\prime \prime }⊕R\left(F_{i}^{\prime }\right)\right),$$

where ⊕ denotes element-wise addition, R(·) represents the residual connection, and $\varphi_{i}$ are learnable channel attention weights. Finally, $F_{\mathrm{temp}}$ are adjusted in the channel dimension to provide a structured and informative feature representation for subsequent tasks.

**Figure 3:**
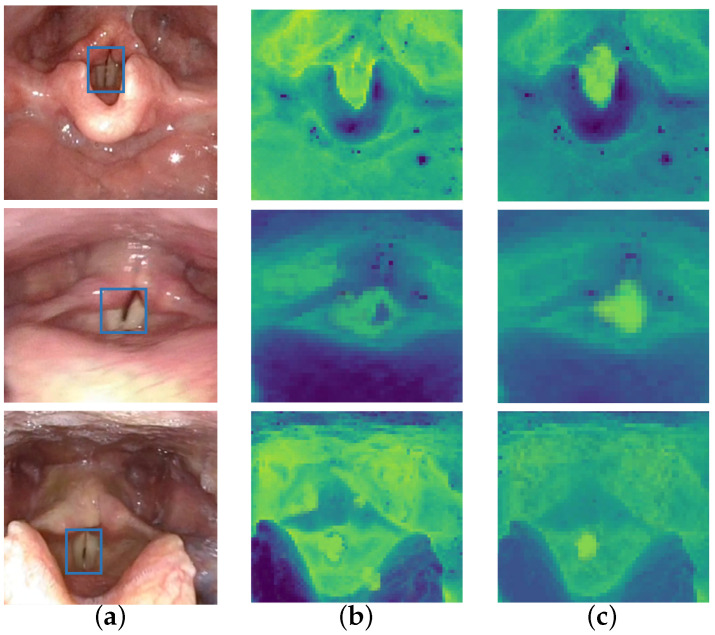
Visual assessment of feature map quality with and without the proposed feature aggregator on clinical data. (**a**) Target region annotations (blue boxes) for each sample. (**b**) Standard feature extraction results. (**c**) Aggregator-enhanced feature extraction results. For the feature maps, the heatmap encodes activation intensity, where warm colors correspond to higher responses and cool colors to lower responses. Compared with standard features, the proposed aggregator suppresses background noise and increases the contrast of the glottal region, producing more compact and localized activations. These results indicate improved semantic focus and spatial discriminability under complex anatomical variations.

This aggregation process effectively integrates multi-scale contextual cues, enabling the model to focus on semantically consistent patterns, such as glottal contours and surrounding anatomical regions. By implicitly encoding semantic priors through channel attention and hierarchical fusion, the module enhances the spatial discriminability and robustness of feature representations under clinical variations, including deformation and lighting fluctuations. To qualitatively validate the effectiveness of the feature aggregation module, we visualize the attention feature maps before and after enabling the module, as shown in Figure 3. The results demonstrate that, after aggregation, attention becomes more concentrated around the glottal region, with enhanced activation intensity and spatial coherence. This confirms that the proposed module successfully amplifies task-relevant semantic signals, thereby improving downstream segmentation quality.

#### 3.3.2. Dual-Path Dynamic Feature Pyramid

Building upon the previously aggregated multi-scale features, we introduce a dual-path dynamic feature pyramid to further enhance hierarchical semantic representation and structural awareness. This module leverages two complementary parameter-adaptive pathways to transform features across multiple resolutions while preserving rich contextual priors. Specifically, each hierarchical level is processed through two parallel paths, an upsampling path that employs transposed convolution to perform learnable interpolation and a downsampling path that applies max pooling to retain high-response local features. These pathways jointly enable the model to capture both fine-grained spatial details and high-level semantic abstractions. We construct a five-level feature pyramid. This design is consistent with the encoder’s hierarchical resolutions and provides sufficient scale granularity to capture both fine glottic structures and broader anatomical context without incurring excessive computation. At each level, we generate an upsampled feature $F_{up}^{j}$ and a downsampled feature $F_{down}^{j}$ and obtain the final representation by dynamically weighted fusion of the two paths:

(6) $$F_{\mathrm{final}}=\sum_{j=1}^{5}\left(\beta_{j}\cdot T\left(F_{up}^{j}\right)+\gamma_{j}\cdot D\left(F_{down}^{j}\right)\right),$$

where T(·) and D(·) represent upsampling via transposed convolution and downsampling via max pooling, respectively. The adaptive weights $\beta_{j}$ and $\gamma_{j}$ are generated by an attention mechanism and satisfy the constraint $\beta_{j}+\gamma_{j}=1$. This fusion strategy adaptively regulates the influence of each pathway according to local feature variations, thereby enhancing the semantic richness and spatial consistency of the resulting feature representation. The design also facilitates stable gradient flow across scales, contributing to improved generalization under complex internal cavity conditions.

#### 3.3.3. Multi-Head Cooperative Feature Decoupling

We utilize region proposal network (RPN) and multi-head perception mechanism to transform the obtained multi-scale semantic features into prompt embeddings required by the SAM mask decoder. The RPN predicts a set of anchor boxes $A={\left\{a_{i}\right\}}_{i=1}^{N}$ at each spatial location of the feature map, utilizing a combination of scales $\left\{s_{1},s_{2},\ldots ,s_{k}\right\}$ and aspect ratios $\left\{r_{1},r_{2},\ldots ,r_{m}\right\}$ to identify potential targets. These candidates are refined via RoI pooling to standardize features into fixed-size vectors, serving as inputs to the subsequent multi-head decoupling stage. The multi-head module consists of three parallel heads: a classification head, a localization head, and a prompt head. The classification head identifies semantic categories, encoding high-level semantic priors. The localization head predicts bounding-box offsets and aligns the prompt with precise spatial locations, capturing spatial priors. The prompt head generates embedding vectors E[i,:] that integrate both visual content and contextual cues for each candidate region, thereby providing semantic guidance for the SAM decoder. These outputs are fused into a unified sparse prompt representation $F_{\mathrm{sparse}}$ through parallel processing. To further enhance spatial awareness, explicit position encoding is introduced, assigning unique spatial representations to each region, which facilitates precise structural alignment and improves downstream mask decoding. A total loss function is defined, which includes the region proposal network loss $L_{rpn}$, classification loss $L_{cls}$, bounding-box regression loss $L_{box}$, and mask loss $L_{mask}$:

(7) $$L=L_{rpn}+L_{cls}+\lambda_{1}L_{box}+\lambda_{2}L_{mask},$$

where $L_{rpn}$ consists of the classification loss and bounding-box regression loss for anchor boxes. The $L_{box}$ is used to optimize the overlap between the predicted bounding box and the ground-truth bounding box, typically computed using the smooth L1 loss. The weights of these losses are controlled by hyperparameters $\lambda_{1}$ and $\lambda_{2}$, which help to balance the impact of the different losses. Finally, the sparse prompt features that are enriched with positional information are fed into the SAM decoder for bilinear interpolation. This process unifies the feature representation space, enabling progressive refinement of prediction results through iterative decoding.

## 4. Experiments

### 4.1. Hardware System

**Figure 4:**
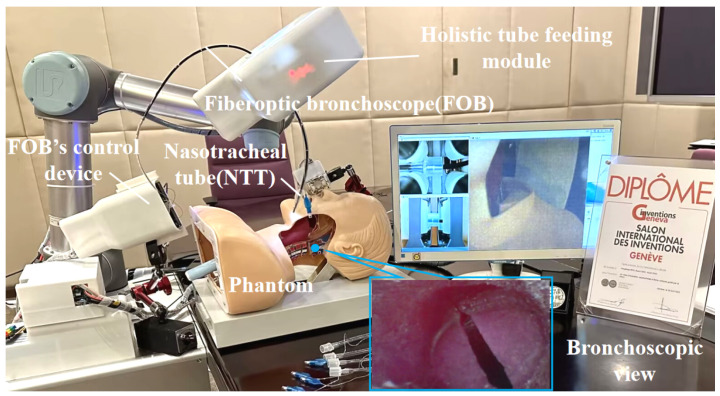
NTI robot system. The system uses flexible robotic arms and end effectors to deliver FOB into the nasal cavity and gradually penetrate the trachea along the calculated path, achieving precise intubation actions.

To comprehensively verify the effectiveness and reliability of the proposed algorithm in real medical application scenarios in Figure 4, we design a NTI robotic system to autonomously and accurately insert a nasotracheal tube (NTT) through the nostrils and thus build a stable and efficient connection between the patient’s airway and the oxygen concentrator. During the testing phase, a robotic arm is used to remotely manipulate the distal portion of the fiberoptic bronchoscope (FOB) according to a predefined bending angle, ensuring that the desired position is achieved with each maneuver. The bronchoscope view, an important part of the system, shows the field of view captured by the camera at the distal tip of the FOB in real time, providing clear and intuitive visual feedback to the operator. Meanwhile, the integral feeder module is responsible for collaboratively controlling the movement of the external components of the FOB and NTT to ensure a smooth and accurate insertion process. The FOB tip is guided from the exterior of the body model into the nostril, and then along the nasal cavity, the pharynx, through the glottic opening, and ultimately into the trachea. These images not only contain the details of key areas, such as the nostrils and nasal passages, but also clearly demonstrate key structures, such as the glottic suture and the right and left glottic valves, which provide invaluable data support for subsequent algorithm optimization and performance evaluation.

### 4.2. Experiments Setup

#### 4.2.1. Dataset

This study uses three datasets to evaluate the effectiveness of our method.

**Figure 5:**
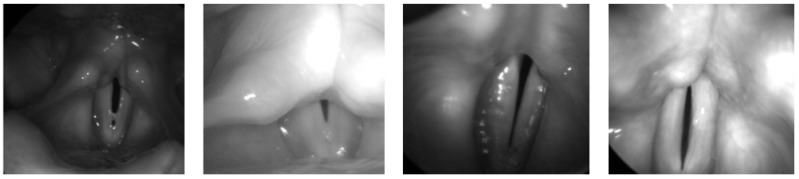
Sample images from the Glottis dataset.

**Figure 6:**
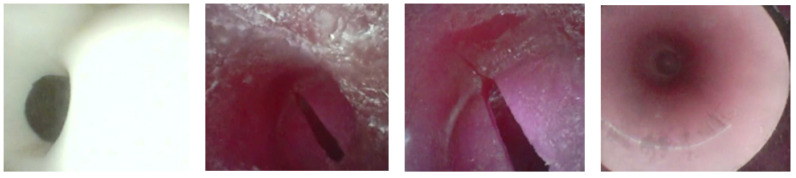
Sample images from the Phantom 2025 dataset.

**Figure 7:**
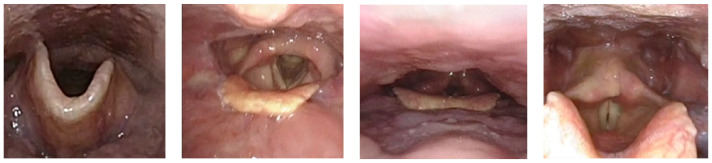
Sample images from the Clinical 2025 dataset.

- Glottis: The Benchmark for Automatic Glottis Segmentation (BAGLS) [35] dataset comprises high-speed videoendoscopic recordings from 640 subjects, including both healthy individuals and patients with various laryngeal disorders. The data are collected by multiple medical professionals using diverse endoscopic systems, introducing substantial heterogeneity in image resolution, lighting conditions, and anatomical variations, as illustrated in Figure 5. To adapt this dataset for object detection tasks, we derive bounding boxes that tightly enclose the original segmentation masks and convert the annotations into the COCO [36] format. The resulting dataset includes 55,750 images for training and 3500 for testing. This large-scale and diverse dataset serves as a critical benchmark for evaluating the generalization capability and real-world applicability of our model across different patient groups and imaging devices.
- Phantom 2025: To mitigate ethical constraints during the initial development stage, we construct a synthetic dataset using our robotic NTI system. The Phantom dataset [37] is collected in a controlled laboratory setting using a fiberoptic bronchoscope equipped with real-time navigation and feedback control, with representative samples shown in Figure 6. The dataset includes 2267 training images and 479 test images. Bounding boxes are used to label general structures, such as the nose, channel, glottis, and trachea. Masks are employed to annotate structures with more clearly defined boundaries or specific shapes, including the right nostril, left nostril, glottic slit, right glottal valve, and left glottal valve, providing pixel-level accurate representations of their form and extent. These samples provide diverse anatomical representations that support early-stage model training and validation without involving human subjects.
- Clinical 2025: The Clinical dataset [37] is obtained from nasopharyngoscopy procedures conducted at the Singapore General Hospital. It comprises 82 high-definition video recordings, each obtained from a unique patient, reflecting a broad spectrum of anatomical diversity and clinically realistic imaging conditions, as exemplified in Figure 7. All procedures are conducted by board-certified otolaryngologists using commercial flexible nasopharyngoscopes to ensure procedural consistency and clinical realism. From these recordings, we extract 2683 training images with 7030 annotated structures and 1131 validation images with 3295 annotations. These images capture key anatomical landmarks, such as the nose, nostrils, epiglottis, glottic valves, and surrounding tissues, as the endoscope passes through the glottis into the trachea.

#### 4.2.2. Implementation Details

We conduct comprehensive ablation and qualitative experiments to evaluate the proposed framework. All experiments were implemented in PyTorch (v1.13.0) with CUDA 11.7 on an NVIDIA GeForce RTX 4090. All models listed in Table 2 are trained for 100 epochs across three datasets. The input images are uniformly resized to a resolution of 256 by 256 pixels. A batch size of 32 is used for the main experiments, while a smaller batch size of 16 is applied during the ablation studies. The training procedure adopts the AdamW optimizer with an initial learning rate of 0.001, a weight decay of 0.05, and a stepwise learning rate scheduling strategy. To ensure a fair comparison, each baseline model is trained using the hyperparameters recommended in its original publication. For reproducibility, all experiments are conducted with fixed random seeds, and model selection is based on the highest validation accuracy achieved during training.

#### 4.2.3. Evaluation Metrics

To comprehensively evaluate the effectiveness of the proposed segmentation method, we adopt a set of evaluation metrics that jointly consider segmentation accuracy and computational efficiency. For segmentation performance evaluation, we adopt the COCO instance segmentation evaluation protocol. The primary metric is mean average precision for masks (mAP), which measures the overall segmentation quality across all classes and confidence thresholds. Additionally, we report ${\mathrm{AP}}_{50}$ and ${\mathrm{AP}}_{75}$, representing average precision at IoU thresholds of 0.5 and 0.75, respectively, where IoU is calculated based on pixel-level mask overlap. These metrics specifically reflect the model’s ability to generate high-quality instance-level masks at low and high matching precision levels. To further evaluate pixel-wise segmentation quality, we incorporate the mean Dice coefficient (mDice), which directly quantifies the spatial overlap between predicted and ground-truth masks. The Dice coefficient for multi-class instance segmentation is calculated as

(8) $$mDice=\frac{1}{n}\sum_{i=1}^{n}\frac{2|P_{i}\cap G_{i}|}{|P_{i}\left|+\right|G_{i}|}$$

where $P_{i}$ and $G_{i}$ represent the predicted and ground-truth binary masks for instance *i*, respectively, and *n* denotes the total number of instances across all classes. This metric is particularly sensitive to boundary precision and complements the AP-based metrics by providing direct pixel-level accuracy assessment. In addition to mDice, we report the Jaccard Index in terms of mean intersection over union mIoU as a complementary overlap-based metric. mIoU measures the overlap between predicted and ground-truth masks via the intersection over union ratio, computed for each instance and then averaged across all instances and classes. Compared with Dice, IoU is typically more sensitive to both over- and under-segmentation, offering an additional view of pixel-level segmentation quality.

For practical deployment considerations, we evaluate computational efficiency through three key metrics. The number of trainable parameters reflects the actual parameters updated during LoRA-based fine-tuning, directly impacting training memory requirements and adaptation efficiency. Inference speed, measured in frames per second (FPS), quantifies real-time processing capability essential for practical applications. Model size in megabytes (MB) reflects the storage requirement and memory usage, which is essential for deployment on devices with limited computational resources. This comprehensive evaluation framework allows for a detailed analysis of the trade-off between accuracy and efficiency in our segmentation method, ensuring both precise mask prediction and practical applicability across various hardware platforms.

### 4.3. Comparison with State-of-the-Art Methods

To comprehensively assess the performance of the proposed Glottis-SAM, we conduct systematic comparative experiments on three public datasets: Glottis, Phantom, and Clinical. The comparison includes traditional CNN-based segmentation models, transformer-based frameworks, and recent architectures derived from SAM, as presented in Table 2. We observe notable performance variations across different datasets when using various methods. Classical CNN-based models, such as Mask R-CNN [11] and PointRend [12], often struggle with distinguishing anatomically similar and morphologically variable structures like the epiglottis and vocal folds. This is largely due to their limited ability to capture global contextual information, resulting in unstable segmentation accuracy. Although more advanced transformer-based models like Mask2Former [38] with Swin-T backbone are introduced, the actual performance improvement remains modest. This suggests that simply increasing model complexity does not necessarily yield proportional gains in segmentation accuracy, particularly in medical imaging tasks. Moreover, lightweight models such as SOLOv2 [18] offer faster inference speed but experience drops in performance, making them inadequate for clinical scenarios that demand high precision. SAM-based model variants exhibit promising performance, demonstrating the potential of foundation models in medical image segmentation, but their large model sizes pose a major obstacle to practical deployment.

#### 4.3.1. Glottis

The FastSAM series further pushes accuracy boundaries, and FastSAM-X [40] reaches the highest mAP, but at the cost of a model size of 868.0 MB and a drastic drop in inference speed to 7.4 FPS, which is unsuitable for resource-limited environments. In contrast, Glottis-SAM demonstrates a significant advantage, striking an optimal balance between accuracy and computational efficiency through LoRA-based fine-tuning. Leveraging a ViT-T backbone, Glottis-SAM achieves 54.3% mAP with a model size of only 55.2 MB, nearly an order of magnitude smaller than most conventional methods. This efficient design makes it particularly suitable for edge computing scenarios.

#### 4.3.2. Phantom

This dataset presents additional challenges, including imaging artifacts and blurry boundaries, which lead to greater performance degradation. Traditional methods such as SparseInst [20] struggle under these conditions, highlighting their limitations in complex visual environments. Even SAM [5] achieves only 40.7% mAP. In contrast, Glottis-SAM obtains a superior mAP of 43.8% by leveraging hierarchical feature adaptation and geometric perception mechanisms, outperforming all compared methods. It also maintains robust segmentation performance under degraded imaging conditions.

#### 4.3.3. Clinical

The Clinical dataset represents the most challenging scenario, encompassing various real-world clinical interferences, such as occlusion, motion blur, and lighting variations, which increase the difficulty of the segmentation. Even YOLACT [15], which performs well on the other two datasets, can only achieve 24.2% mAP. Large models like FastSAM also struggle to maintain stable performance. Our analysis attributes this to the rigidity of their feature extraction mechanisms, which lack adaptability to real-world environmental variations. Glottis-SAM achieves stable performance under various interference conditions, demonstrating the robustness of our proposed feature aggregation in handling complex medical imaging tasks.

In summary, Glottis-SAM consistently outperforms state-of-the-art methods across all datasets, demonstrates superior accuracy, and shows strong robustness to real-world variability. These advantages highlight its strong potential for deployment in practical medical scenarios, particularly on resource-constrained devices such as portable and embedded systems.

### 4.4. Ablation Study

#### 4.4.1. Low-Rank Aaptation

A well-chosen scope for LoRA layers can reduce model size while maintaining strong performance. We investigate how different LoRA deployment strategies across network modules affect overall performance. As shown in Table 3, the model achieves mDice of 66.9 without any LoRA layers. When LoRA is applied solely to the QKV attention matrices, mAP increases from the baseline 40.7% to 43.3%, indicating that introducing low-rank structures into attention computation helps to extract effective features. We further expand the application to the feedforward layers of the MLP, integrating LoRA in both $Fc_{1}$ and $Fc_{2}$. When QKV, $Fc_{1}$, and $Fc_{2}$ adaptations are jointly enabled, the model achieves optimal balance, with mDice improving to 70.5%. This configuration maximizes the feature extraction and representation capability of the model.

#### 4.4.2. Rank and Scaling Factor

The low-rank constraint helps to suppress redundant feature interference, while the scaling factor enhances the representation of glottal microstructures. We investigate the synergistic effects of LoRA on the performance under various rank *r* and scaling factor α configurations. In Table 4, when *r* increases from 2 to 64, model performance first improves and then declines. Although a higher rank enables the capture of more high-frequency details, it may also introduce noise, thereby impairing segmentation accuracy. With r=4 fixed, the configuration with α=32 achieves a higher mAP than α=64 despite having only 0.15 M trainable parameters. This confirms the beneficial effect of a moderate scaling factor in enhancing model generalization. Specifically, when α=32 and r=4, the model achieves well-balanced performance on the Phantom dataset, with an mAP of 42.7% and an ${\mathrm{AP}}_{50}$ of 67.2%, significantly outperforming other configurations. This setting is thus identified as the optimal configuration for glottis segmentation.

#### 4.4.3. Channels in FPN

To investigate the influence of hyperparameter settings on network performance, we conduct an ablation study on the number of channels to evaluate the impact on model performance. In Table 5, reducing the number of channels from 256 to 64 leads to a significant improvement in key performance metrics while maintaining real-time inference speed. When the channel is set to 64, the model achieves its highest accuracy, with an ${\mathrm{AP}}_{50}$ of 67.2%. This improvement can be attributed to the fine-grained anatomical structures of the glottis, where an excessive number of channels may introduce redundant noise, whereas moderate channel compression helps to enhance focus on salient edge features. However, when the number is further reduced to 32, the mAP declines due to the loss of shallow semantic information. An extreme reduction to 8 channels causes a dramatic drop in mAP, leading to feature collapse. These findings suggest that 64 channels represent the optimal configuration for glottis segmentation.

#### 4.4.4. Weight of the Loss Function

We adjust the weight parameters $\lambda_{1}$ and $\lambda_{2}$ in the loss function to evaluate the impact of $L_{box}$ and $L_{mask}$ on model performance. As shown in Table 6, the model achieves the best performance when $\lambda_{1}$ is 0.5 and $\lambda_{2}$ is 2, reaching mAP of 44.7%, with ${\mathrm{AP}}_{50}$ and ${\mathrm{AP}}_{75}$ of 71.3% and 51.9%, respectively. Meanwhile, the mDice coefficient for the mask also reaches its highest value of 74.4%. In contrast, increasing $\lambda_{1}$ or decreasing $\lambda_{2}$ leads to performance degradation across all metrics to varying degrees. These findings indicate that, in our task, placing relatively more emphasis on the $L_{mask}$ and appropriately reducing the weight of the $L_{box}$ is beneficial for improving model performance. Although the model size fluctuates slightly between 410 MB and 427 MB under different weight configurations, this variation has minimal impact on performance.

#### 4.4.5. Qualitative Results

**Figure 8:**
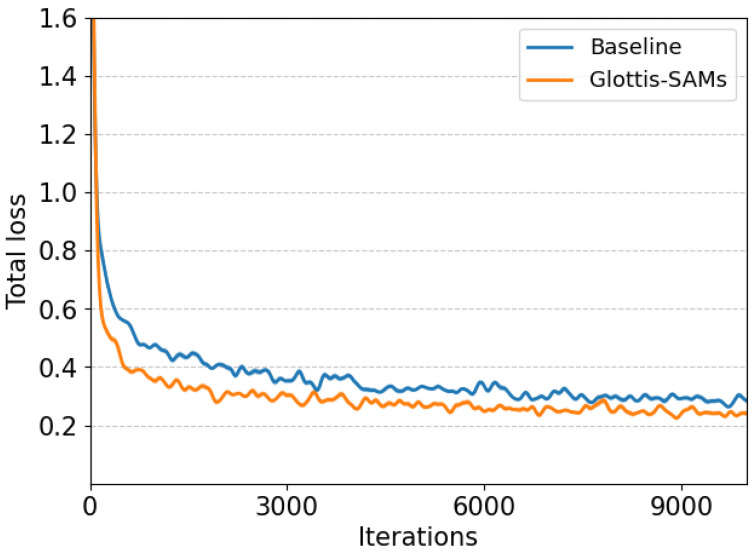
Comparative analysis of total loss during training on the Clinical dataset reveals that our method achieves faster convergence than the baseline approach, indicating more efficient optimization towards the global optimum. This improvement stems from the synergistic effect of our rank-constrained adaptation and dynamic feature pyramid for multi-scale feature learning, while the channel attention mechanism effectively reduces gradient conflicts during hierarchical feature aggregation.

Figure 8 presents the training loss curve on the Phantom dataset, where our model exhibits strong convergence stability throughout the optimization process. The integrated channel attention mechanism helps to alleviate gradient interference during hierarchical feature fusion, while the proposed loss weighting scheme ensures a balanced focus between bounding-box regression and mask accuracy.

**Figure 9:**
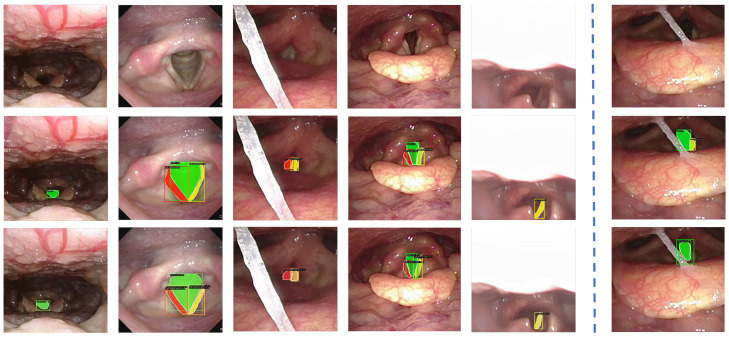
Instance analysis results of our method. The visualization consists of three rows: clinical images (**top**), ground-truth annotations (**middle**), and instance predictions (**bottom**). Different colors denote different categories in the annotations and predictions. The first five columns present successful cases where our method robustly handles challenging NTI scenarios, glottal deformation, motion blur, and lighting variability by leveraging dynamic feature representation and multi-prior contextual modeling for precise parsing from local structures to global semantics. The last column depicts a failure case under severe occlusion, suggesting directions for future improvement via occlusion-aware learning and dataset augmentation.

Figure 9 illustrates segmentation examples under challenging clinical conditions, including lighting variation, anatomical differences, and interference from saliva. Driven by dynamic feature representation and the incorporation of multiple priors, our model consistently achieves precise glottis localization and maintains robust mask quality despite background complexity. Nevertheless, failure cases occasionally occur when the glottal region is severely occluded by anatomical tissue, which limits the effectiveness of current sparse prompt embeddings and leads to incomplete detection or inaccurate segmentation. These observations highlight the need for more robust prompt decoding and feature reasoning under extreme conditions.

## 5. Discussion

We present Glottis-SAM, a foundation model adaptation framework for glottis segmentation in NTI. The framework combines dynamic multi-scale feature aggregation with multi-prior guidance and uses a rank-constrained parameter adaptation strategy. Together, these designs help the model to focus on the glottal structure under complex anatomy, illumination changes, and motion blur that are common in endoscopic scenes.

From a broader perspective, Glottis-SAM offers a practical path to adapt general foundation models to structure-centric medical segmentation. Rank-constrained adaptation supports efficient transfer with limited training cost, which is valuable when computational resources are constrained or when fast deployment across devices is required. Meanwhile, the joint use of multi-prior guidance and multi-scale aggregation encourages consistent boundary awareness and improves the preservation of fine-grained structural cues, which is critical for small and deformable targets such as the glottis.

There are still challenging cases. Severe occlusions by surrounding tissues or reflective fluids can reduce the reliability of sparse prompts and lead to partial masks. In addition, the current study focuses on static images and does not yet benefit from temporal continuity in endoscopic videos, which could further stabilize predictions and reduce frame-to-frame jitter. These observations motivate future work on occlusion-aware decoding, stronger robustness-oriented data synthesis, and temporally consistent video segmentation, together with broader validation across clinical centers and endoscopic devices.

## 6. Conclusions

Glottis-SAM provides an effective and deployable solution for glottis segmentation in transnasal intubation. Across three datasets, it delivers strong segmentation accuracy while remaining compact and fast enough for real-time use. With a ViT-T backbone, it reaches an mDice of 94.7 and an mIoU of 89.9 on the Glottis dataset, and an mDice of 72.6 and an mIoU of 57.0 on the Clinical dataset. It also supports compact and fast inference, with a model size of 55.2 MB and an inference speed of 44.3 FPS. These results indicate that Glottis-SAM can serve as a reliable component for real-time endoscopic guidance and provide a solid basis for future extensions toward video-level understanding and more robust performance under extreme visual disturbances. In future work, we will incorporate temporal modeling to leverage frame continuity in endoscopic videos, aiming to improve stability and reduce prediction jitter in challenging scenes. We will also perform broader multi-center validation across diverse endoscopic devices to further assess generalization in real clinical settings.

## Figures and Tables

**Table 1:** An elastic base architecture of ViT. The input image size is 256 × 256.

Stage	Block	Configuration	Output Size
1	MBConv	$\left[\begin{array}{c} \mathrm{embed}\;\dim\;\zeta_{1},\\ \mathrm{expansion}\;\mathrm{ratio}\;4 \end{array}\right]$ × 2	56 × 56
2	Transformer	$\left[\begin{array}{c} \mathrm{embed}\;\dim\;\zeta_{2},\;\mathrm{head}\;\zeta_{2}/32\\ \mathrm{window}\;\mathrm{size}\;7\;\times \;7,\\ \mathrm{mlp}\;\mathrm{ratio}\;4 \end{array}\right]$ × 2	28 × 28
3	Transformer	$\left[\begin{array}{c} \mathrm{embed}\;\dim\;\zeta_{3},\;\mathrm{head}\;\zeta_{3}/32\\ \mathrm{window}\;\mathrm{size}\;14\;\times \;14,\\ \mathrm{mlp}\;\mathrm{ratio}\;4 \end{array}\right]$ × 6	14 × 14
4	Transformer	$\left[\begin{array}{c} \mathrm{embed}\;\dim\;\zeta_{4},\;\mathrm{head}\;\zeta_{4}/32\\ \mathrm{window}\;\mathrm{size}\;7\;\times \;7,\\ \mathrm{mlp}\;\mathrm{ratio}\;4 \end{array}\right]$ × 2	7 × 7

**Table 2:** Comparison of accuracy with the state-of-the-art methods: Glottis, Phantom, and Clinical datasets are evaluated in terms of mAP, AP_50_, mDice, mIoU, model size (MB), and inference speed (FPS).

Models	Backbones	Glottis Dataset				Phantom Dataset				Clinical Dataset				Model Size (MB)	Infer (FPS)
		mAP	AP_50_	mDice	mIoU	mAP	AP_50_	mDice	mIoU	mAP	AP_50_	mDice	mIoU		
BoxInst [16]	ResNet101	10.6	31.8	34.4	20.8	25.1	45.2	64.1	47.2	8.8	27.1	33.0	19.8	447.3	45.7
CondInst [17]	ResNet50	13.5	39.1	43.9	28.1	31.0	55.4	77.1	62.7	13.2	36.5	43.3	27.6	279.2	46.8
Mask-RCNN [11]	ResNet101	44.7	79.0	79.6	66.1	39.7	65.6	70.4	54.3	30.1	55.3	66.8	50.2	506.3	42.2
Mask-RCNN [11]	Swin-T	47.1	82.9	83.5	71.7	40.0	64.9	67.8	51.3	28.3	53.1	65.0	48.1	572.0	38.2
MS-RCNN [13]	X101	45.5	78.1	79.0	65.3	31.3	57.0	62.9	45.9	25.5	50.0	60.2	43.1	634.4	43.0
PointRend [12]	ResNet50	45.1	81.8	82.9	70.8	40.5	64.4	65.2	48.4	30.9	55.7	66.5	49.8	447.3	41.4
Mask2Former [38]	ResNet101	37.6	79.7	83.0	70.9	36.7	56.9	72.9	57.4	23.4	52.1	59.8	42.7	787.1	19.8
Mask2Former [38]	Swin-T	34.5	76.5	80.1	66.8	34.0	53.3	61.9	44.8	28.3	53.1	65.0	48.1	599.6	18.8
QueryInst [14]	ResNet50	42.2	84.9	89.2	80.5	18.7	33.8	45.5	29.4	15.6	32.6	44.5	28.6	208.7	30.8
RTMDet [19]	CSPDarkNet	42.4	83.2	86.5	76.2	23.0	39.6	42.3	26.8	19.3	40.6	50.7	34.0	977.7	37.2
U-Net++ [21]	–	–	–	78.3	64.3	–	–	69.3	53.0	–	–	49.6	33.0	32	55.6
U-Net3+ [22]	–	–	–	77.3	63.0	–	–	54.0	37.0	–	–	35.9	21.9	104	33.3
SOLOv2 [18]	ResNet50	22.5	57.0	58.2	41.0	13.3	37.0	32.7	19.5	4.6	17.4	25.8	14.8	371.6	45.9
SparseInst [20]	ResNet50	52.1	89.1	90.6	82.8	28.5	44.6	51.4	34.6	19.7	43.5	48.3	31.8	418.5	69.9
YOLACT [15]	ResNet101	48.7	90.4	91.5	84.3	40.9	66.7	71.4	55.5	24.2	50.2	61.3	44.2	460.8	72.8
RSPrompter [39]	ViT-B	14.6	39.6	45.5	29.4	15.1	31.7	33.4	20.0	5.4	15.1	25.4	14.5	470.0	12.2
SAM [5]	ViT-B	52.7	90.4	92.7	86.4	40.7	64.3	67.2	50.6	28.2	50.5	64.6	47.7	441.5	12.8
FastSAM-S [40]	CSPDarkNet	56.9	92.7	–	–	39.4	59.8	–	–	27.5	46.2	–	–	148.5	25.9
FastSAM-X [40]	CSPDarkNet	**57.2**	93.5	–	–	35.1	55.5	–	–	26.5	43.4	–	–	868.0	7.4
Glottis-SAM (Ours)	ViT-B	53.8	92.0	93.0	86.9	44.7	71.3	74.4	59.2	28.8	55.0	65.7	48.9	392.6	13.2
Glottis-SAM (Ours)	ViT-T	55.8	93.6	94.7	89.9	43.8	66.8	73.1	57.6	34.4	63.2	72.6	57.0	55.2	44.3

**Table 3:** Comparison of LoRA fine-tuning introduced in different adapter layers: the query–key–value (QKV) projection matrices and the two fully connected layers ($FC_{1}$ and $FC_{2}$) of the feedforward network. ✓ indicates that LoRA is applied to the corresponding layer, while × indicates that it is not applied.

*QKV*	*FC*_1_	*FC*_2_	mAP	AP_50_	AP_75_	mDice	Model Size (MB)
×	×	×	40.7	64.3	49.2	66.9	445.2
✓	×	×	43.3	66.1	49.7	69.6	417.1
✓	✓	×	42.3	65.1	49.0	67.8	417.5
✓	×	✓	42.9	65.4	50.9	68.2	414.6
✓	✓	✓	43.0	67.4	51.0	70.5	414.1

**Table 4:** Impact of scaling factor α and rank *r* in LoRA fine-tuning on glottis segmentation performance.

*α*	*r*	mAP	AP_50_	AP_75_	mDice	Trainable Params (M)
64	2	38.8	63.1	44.8	66.7	0.07
	4	40.1	63.9	44.5	68.1	0.15
	8	41.2	65.5	47.9	70.1	0.29
	16	41.8	66.6	48.1	69.8	0.59
	32	40.8	66.3	45.9	68.7	1.38
	64	42.6	65.6	49.5	69.6	2.36
32	2	41.5	64.8	47.9	68.1	0.07
	4	42.7	67.4	48.4	70.5	0.15
	8	41.3	65.6	44.2	69.1	0.29
	16	41.9	66.7	47.1	69.9	0.59
	32	41.6	66.3	47.1	69.6	1.38
	64	41.3	66.3	45.9	67.9	2.36

**Table 5:** Performance of FPN with various channel numbers on the Phantom dataset.

Channels	mAP	AP_50_	AP_75_	mDice	Infer (FPS)
256	41.8	66.8	46.6	70.3	11.40
128	40.1	64.3	45.0	67.5	11.47
64	42.5	67.2	48.6	70.7	12.16
32	41.7	65.2	46.1	68.7	12.52
16	38.6	60.2	42.4	67.1	12.54
8	16.5	36.5	11.9	44.9	12.87

**Table 6:** Performance of $L_{\mathrm{box}}$ and $L_{\mathrm{mask}}$ with various weights on the Glottis dataset.

*λ*_1_	*λ*_2_	mAP	AP_50_	AP_75_	mDice	Model Size (MB)
0.5	2	44.7	71.3	51.9	74.4	421.3
1	0.5	41.7	64.3	47.9	67.9	427.0
1	1	43.0	67.8	50.9	73.3	414.7
1	2	43.6	66.9	50.8	70.2	412.2
2	0.5	41.2	64.3	48.5	68.1	410.5

## Data Availability

Our experimental evaluation was conducted on three publicly available datasets. The BAGLS dataset can be accessed via Zenodo (accessed on 12 February 2025; https://doi.org/10.5281/zenodo.3377544) and Kaggle (accessed on 12 February 2025; https://www.kaggle.com/datasets/gomezp/benchmark-for-automatic-glottis-segmentation). The Phantom and Clinical datasets are publicly available on Scientific Data (accessed on 9 December 2025; https://doi.org/10.1038/s41597-025-06169-0) with open access.

## References

[1] Zhu Z., Wang Z., Qi G., Mazur N., Yang P., Liu Y. (2024). Brain tumor segmentation in MRI with multi-modality spatial information enhancement and boundary shape correction. Pattern Recognit..

[2] Liu H., Liu S.Q., Xie X.L., Li Y., Zhou X.H., Feng Z.Q., Li G.T., Ma X.Y., Hou Z.G., Yuan Y.Y. (2023). An Original Design of Robotic-Assisted Flexible Nasotracheal Intubation System. 2023 IEEE International Conference on Robotics and Biomimetics (ROBIO).

[3] Zheng L., Wu H., Yang L., Lao Y., Lin Q., Yang R. (2020). A Novel Respiratory Follow-up Robotic System for Thoracic-Abdominal Puncture. IEEE Trans. Ind. Electron..

[4] Gloger O., Lehnert B., Schrade A., Völzke H. (2014). Fully automated glottis segmentation in endoscopic videos using local color and shape features of glottal regions. IEEE Trans. Biomed. Eng..

[5] Kirillov A., Mintun E., Ravi N., Mao H., Rolland C., Gustafson L., Xiao T., Whitehead S., Berg A.C., Lo W.Y. Segment Anything. Proceedings of the IEEE/CVF International Conference on Computer Vision.

[6] Huang Y., Yang X., Liu L., Zhou H., Chang A., Zhou X., Chen R., Yu J., Chen J., Chen C. (2024). Segment Anything Model for Medical Images. Med. Image Anal..

[7] Cerrolaza J.J., Osma-Ruiz V., Sáenz-Lechón N., Villanueva A., Gutiérrez-Arriola J.M., Godino-Llorente J.I., Cabeza R. (2011). Fully-automatic glottis segmentation with active shape models. MAVEBA.

[8] Karakozoglou S., Henrich N., d’Alessandro C., Stylianou Y. (2012). Automatic Glottal Segmentation Using Local-Based Active Contours and Application to Glottovibrography. Speech Commun..

[9] Fehling M.K., Grosch F., Schuster M.E., Schick B., Lohscheller J. (2020). Fully Automatic Segmentation of Glottis and Vocal Folds in Endoscopic Laryngeal High-Speed Videos Using a Deep Convolutional LSTM Network. PLoS ONE.

[10] Deng K., Tao B., Hu J. (2024). Unpaired Medical Image Enhancement Based on Generative Adversarial Networks. Fifteenth International Conference on Information Optics and Photonics.

[11] He K., Gkioxari G., Dollár P., Girshick R. Mask R-CNN. Proceedings of the IEEE International Conference on Computer Vision (ICCV).

[12] Kirillov A., Wu Y., He K., Girshick R. PointRend: Image Segmentation as Rendering. Proceedings of the IEEE/CVF Conference on Computer Vision and Pattern Recognition (CVPR).

[13] Huang Z., Huang L., Gong Y., Huang C., Wang X. Mask Scoring R-CNN. Proceedings of the IEEE Conference on Computer Vision and Pattern Recognition (CVPR).

[14] Fang Y., Yang S., Wang X., Li Y., Fang C., Shan Y., Feng B., Liu W. Instances as Queries. Proceedings of the IEEE International Conference on Computer Vision.

[15] Bolya D., Zhou C., Xiao F., Lee Y.J. Yolact: Real-time instance segmentation. Proceedings of the IEEE/CVF International Conference on Computer Vision.

[16] Tian Z., Shen C., Wang X., Chen H. BoxInst: High-Performance Instance Segmentation with Box Annotations. Proceedings of the IEEE/CVF Conference on Computer Vision and Pattern Recognition (CVPR).

[17] Tian Z., Shen C., Chen H. (2020). Conditional Convolutions for Instance Segmentation. European Conference on Computer Vision (ECCV).

[18] Wang X., Zhang R., Kong T., Li L., Shen C. (2020). SOLOv2: Dynamic and fast instance segmentation. Adv. Neural Inf. Process. Syst..

[19] Lyu C., Zhang W., Huang H., Zhou Y., Wang Y., Liu Y., Zhang S., Chen K. (2022). RTMDet: An Empirical Study of Designing Real-Time Object Detectors. arXiv.

[20] Cheng T., Wang X., Chen S., Zhang W., Zhang Q., Huang C., Zhang Z., Liu W. Sparse Instance Activation for Real-Time Instance Segmentation. Proceedings of the IEEE/CVF Conference on Computer Vision and Pattern Recognition (CVPR).

[21] Zhou Z., Rahman Siddiquee M.M., Tajbakhsh N., Liang J. (2018). Unet++: A nested u-net architecture for medical image segmentation. Deep Learning in Medical Image Analysis and Multimodal Learning for Clinical Decision Support: 4th International Workshop, DLMIA 2018, and 8th International Workshop, ML-CDS 2018, Held in Conjunction with MICCAI 2018, Granada, Spain, September 20, 2018, Proceedings 4.

[22] Huang H., Lin L., Tong R., Hu H., Zhang Q., Iwamoto Y., Han X., Chen Y.W., Wu J. UNet 3+: A Full-Scale Connected UNet for Medical Image Segmentation. Proceedings of the IEEE International Conference on Acoustics, Speech and Signal Processing (ICASSP).

[23] Derdiman Y.S., Koc T. (2021). Deep learning model development with U-net architecture for glottis segmentation. 2021 29th Signal Processing and Communications Applications Conference (SIU).

[24] Chen X., Liu Q., Deng H.H., Kuang T., Lin H.H.Y., Xiao D., Gateno J., Xia J.J., Yap P.T. (2024). Improving image segmentation with contextual and structural similarity. Pattern Recognit..

[25] Huang Z., Cheng S., Wang L. (2024). Medical image segmentation based on dynamic positioning and region-aware attention. Pattern Recognit..

[26] Cao G., Sun Z., Wang C., Geng H., Fu H., Yin Z., Pan M. (2024). RASNet: Renal automatic segmentation using an improved U-Net with multi-scale perception and attention unit. Pattern Recognit..

[27] Ma J., He Y., Li F., Han L., You C., Wang B. (2024). Segment Anything in Medical Images. Nat. Commun..

[28] Silva-Rodríguez J., Dolz J., Ayed I.B. (2023). Towards foundation models and few-shot parameter-efficient fine-tuning for volumetric organ segmentation. International Conference on Medical Image Computing and Computer-Assisted Intervention.

[29] Wen J., Qin F., Du J., Fang M., Wei X., Chen C.L.P., Li P. (2023). Msgfusion: Medical semantic guided two-branch network for multimodal brain image fusion. IEEE Trans. Multimed..

[30] Dai Z., Yi J., Yan L., Xu Q., Hu L., Zhang Q., Li J., Wang G. (2023). PFEMed: Few-shot medical image classification using prior guided feature enhancement. Pattern Recognit..

[31] Zhao X., Zhang P., Song F., Ma C., Fan G., Sun Y., Feng Y., Zhang G. (2022). Prior attention network for multi-lesion segmentation in medical images. IEEE Trans. Med. Imaging.

[32] Shi H., Han S., Huang S., Liao Y., Li G., Kong X., Liu S. (2024). Mask-enhanced segment anything model for tumor lesion semantic segmentation. International Conference on Medical Image Computing and Computer-Assisted Intervention.

[33] You X., He J., Yang J., Gu Y. (2024). Learning with explicit shape priors for medical image segmentation. IEEE Trans. Med. Imaging.

[34] Hu E.J., Shen Y., Wallis P., Allen-Zhu Z., Li Y., Wang S., Wang L., Chen W. (2022). LoRA: Low-Rank Adaptation of Large Language Models. Int. Conf. Learn. Represent..

[35] Gómez P., Kist A.M., Schlegel P., Berry D.A., Chhetri D.K., Dürr S., Echternach M., Johnson A.M., Kniesburges S., Kunduk M. (2020). BAGLS, a Multihospital Benchmark for Automatic Glottis Segmentation. Sci. Data.

[36] Lin T.Y., Maire M., Belongie S., Hays J., Perona P., Ramanan D., Dollár P., Zitnick C.L. (2014). Microsoft COCO: Common Objects in Context. European Conference on Computer Vision (ECCV).

[37] Hao R., Lai J., Zhong W., Xie D., Tian Y., Zhang T., Zhang Y., Chan C.P.L., Chan J.Y.K., Ren H. (2025). Variable-stiffness nasotracheal intubation robot with passive buffering: A modular platform in mannequin studies. 2025 IEEE International Conference on Robotics and Automation (ICRA).

[38] Cheng B., Misra I., Schwing A.G., Kirillov A., Girdhar R. Masked-Attention Mask Transformer for Universal Image Segmentation. Proceedings of the IEEE/CVF Conference on Computer Vision and Pattern Recognition (CVPR).

[39] Chen K., Liu C., Chen H., Zhang H., Li W., Zou Z., Shi Z. (2024). RSPrompter: Learning to Prompt for Remote Sensing Instance Segmentation Based on Visual Foundation Model. IEEE Trans. Geosci. Remote Sens..

[40] Zhao X., Ding W., An Y., Du Y., Yu T., Li M., Tang M., Wang J. (2023). Fast Segment Anything. arXiv.

